# Recurrent Adenocarcinoma of Colon Presenting as Duodenal Metastasis With Partial Gastric Outlet Obstruction: A Case Report With Review of Literature

**DOI:** 10.4021/wjon624w

**Published:** 2013-05-06

**Authors:** Parag Brahmbhatt, Jason Ross, Atif Saleem, Jason McKinney, Pranav Patel, Sarah Khan, Chakradhar M. Reddy, Mark Young

**Affiliations:** a Department of Internal Medicine, East Tennessee State University, Johnson City, TN 37614, USA; b Division of Gastroenterology and Hepatology, East Tennessee State University, Johnson City, TN 37614, USA

**Keywords:** Recurrent adenocarcinoma of colon, Duodenal metastasis, Gastric outlet obstruction

## Abstract

Colorectal cancer is one of the leading causes of cancer related deaths in western world. While most common site for metastasis for colon cancer is liver, lung, and the peritoneum, metastasis to various other organs such as brain, bones and thyroid has been reported. Metastatic lesions to the small bowel are more common than primary lesions and most common primary neoplasms that metastasize to the duodenum are lung cancer, renal cell carcinoma, breast cancer, and malignant melanoma. We report a very rare case of recurrent adenocarcinoma of colon metastasizing to duodenum after 2 years of curative resection of primary cancer. Surgical resection for curative intent as well as palliative management is recommended.

## Introduction

Although small intestine is the longest part of the digestive tract involving about 75% of total length, incidence of cancer involving it is very low. With the global incidence of about 1.0 per 100,000 populations, malignant neoplasm of small bowel is rare. Adenocarcinoma consists of about 30-40% of all cancers of small bowel and most of the tumors located in duodenum and duodeno-jejunal junction.

According to a study, small intestinal malignancy accounts for only 1-2% of all malignancy of the gastrointestinal tract and accounts for only 1% deaths related to gastrointestinal malignancies [1, 2]. In United States, demographically, black population is the predominantly affected group with higher incidence as well as higher mortality rate [3]. Metastatic lesions to the small bowel are more common than primary lesions and most common primary neoplasms that metastasize to the duodenum are lung cancer, renal cell carcinoma, breast cancer, and malignant melanoma [4, 5].

It is estimated that about 15 to 20 % of patients with colorectal cancer present with metastasis and about 50 to 60 % of patients develops metastasis during the course of their disease [6]. The most common sites of metastasis from colon cancer are the regional lymph nodes, the liver, the lung, and the peritoneum. Occasional cases of metastasis to bone, brain, thyroid and adrenals have been reported in literatures [7, 8]. We report a very rare case of recurrent adenocarcinoma of colon metastasizing to duodenum after 2 years of curative resection of primary cancer.

## Case Report

A 54-year-old woman presented with one week duration of persistent nausea and vomiting in March 2012. Prior to current presentation, patient has experienced ongoing nausea, lasting more than a month with associated symptoms of early satiety and 10 pound weight loss.

Patient’s significant past medical history includes diagnosis of stage IIIC ileocecal adenocarcinoma in December 2009, after being presented with intermittent bowel obstruction. Staging at the time of initial diagnosis did not identify any metastasis. Patient underwent a right hemicolectomy with curative intent and also completed 12 cycles of adjuvant chemotherapy with FOLinic acid-Fluororuracil-OXaliplatin (FOLFOX) regimen in August 2010. Subsequently patient had a normal surveillance workup which included carcinoembryonic antigen (CEA) level, colonoscopy and computerized tomographic (CT) scan in August of 2011 showing no evidence of disease recurrence.

**Figure 1:**
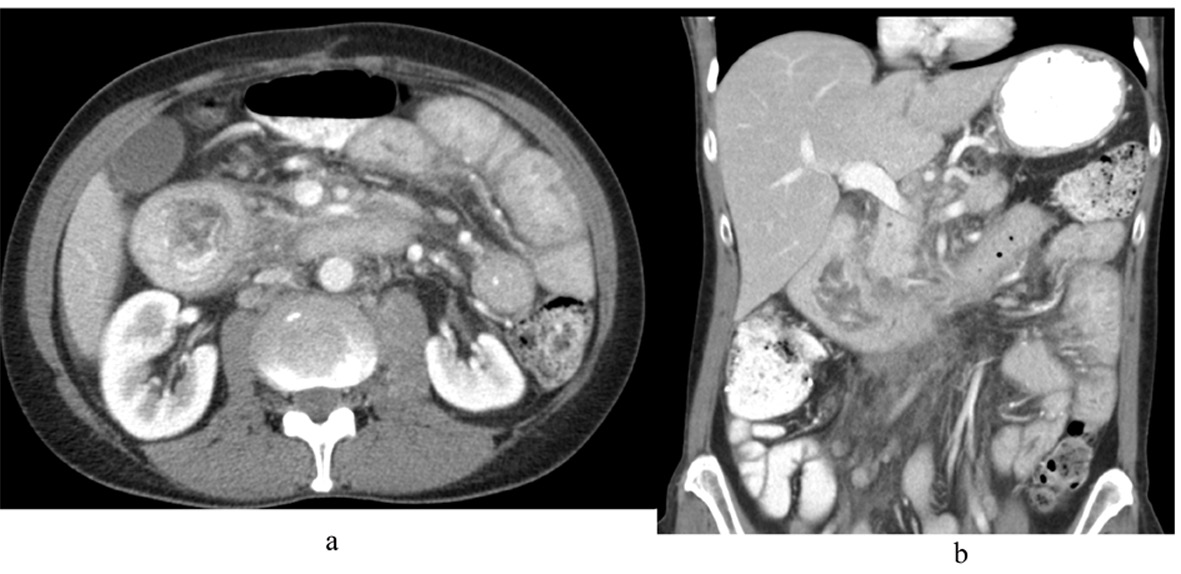
CT scan of abdomen and pelvis with IV contrast (A) axial view and (B) coronal view showing marked distention with irregular wall thickening of the duodenum just proximal to the genu causing a partial gastric outlet obstruction.

During the current presentation, CT scan of abdomen and pelvis with intravenous (IV) contrast revealed marked distention with irregular wall thickening of the duodenum just proximal to the genu causing a partial gastric outlet obstruction (Fig. 1). It also showed enlarged lymph nodes within the small bowel mesentery as well as the retroperitoneum, concerning for recurrent malignant disease.

**Figure 2:**
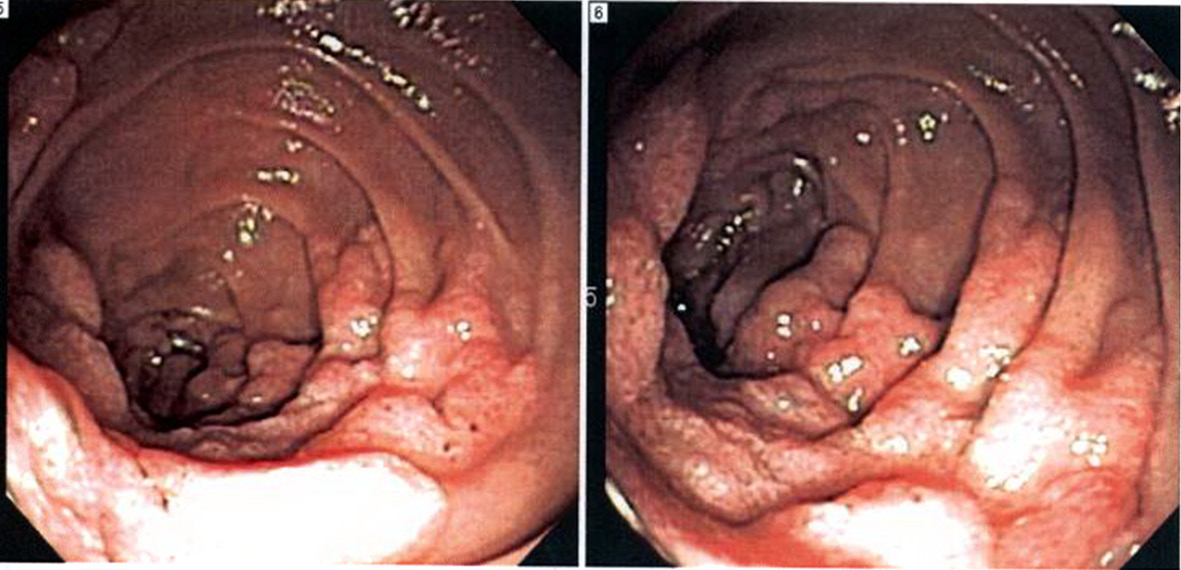
Esophagogastroduodenoscopy (EGD) showing exophytic mass covering 3 quarters of the circumference of the duodenal wall at the second portion of the duodenum.

**Figure 3:**
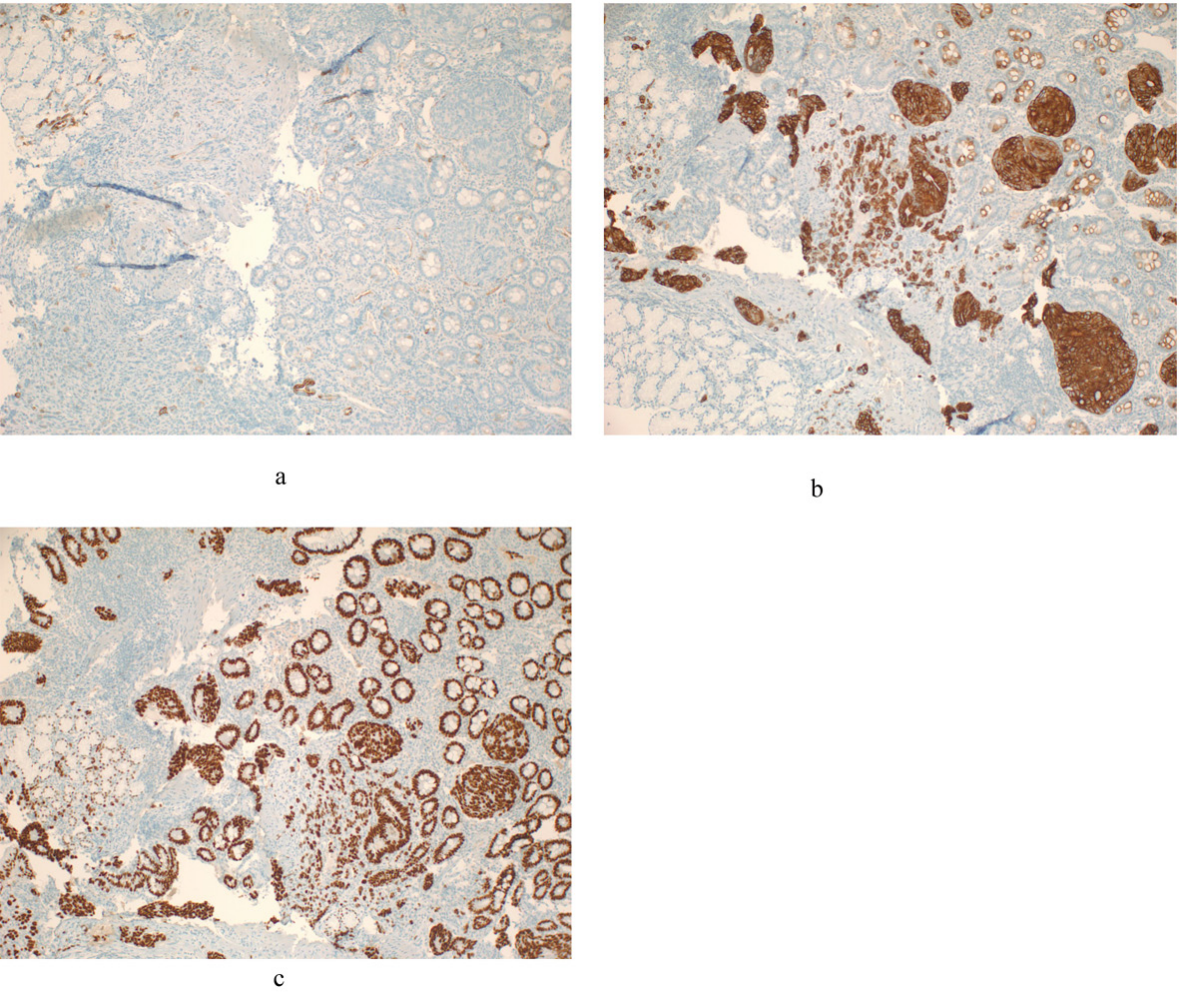
An immunohistochemical staining profile of duodenal biopsy showing CK-7 negative and CK-20 and CD-X2 strongly positive, supporting diagnosis of metastatic colon as the primary neoplasm. A). Cytokeratins 7 (CK-7) negative; B). Cytokeratins 20 (CK-20) positive; C). CD X2 positive.

Patient was hospitalized and an esophagogastroduodenoscopy (EGD) was performed which showed exophytic mass covering 3 quarters of the circumference of the duodenal wall at the second portion of the duodenum with luminal narrowing but no obstruction (Fig. 2). Biopsy specimen of the mass was identified as a moderately differentiated adenocarcinoma and an immunohistochemical staining profile showed CK-7 negative and CK-20 and CD-X2 strongly positive, supporting diagnosis of colon as the primary neoplasm (Fig. 3).

A Positron Emission Tomography (PET) scan revealed development of bilateral metastatic lung disease, metastatic bone disease in the cervical and thoracic spine, adenopathy in the mediastinum, right retrocrural and left iliac regions. A Magnetic Resonance Imaging (MRI) of the brain also showed three 7 mm enhancing nodules in the frontal and parietal lobes. Patient subsequently underwent a palliative gastrojejunostomy to bypass the obstruction caused by the duodenal mass and received palliative brain radiation therapy, but chemotherapy was not started due to poor functional and nutritional status. Patient was then placed on hospice care.

## Discussion

While primary duodenal adenocarcinoma is a rare malignancy, metastasis from a primary colon adenocarcinoma is even less common. About 45% of the all duodenal neoplasia are located in the third and fourth portion of the duodenum, 40% in the second part and only about 15% are located in the first part of the duodenum [1]. Recurrence of colorectal cancer is documented in about 30 to 40% of patients after primary curative surgical resection and majority of these recurrences are seen in first 2 years after surgery [7]. The most common sites of metastasis from colon cancer are the regional lymph nodes, the liver, the lung, and the peritoneum. Occasional cases of metastasis to bone, brain, thyroid and adrenals have been reported in literatures [7, 8].

There is also an association between colorectal cancer and cancer of the small bowel, and presence of one cancer increases the risk of another cancer [9]. According to the report published by the Surveillance, Epidemiology and End Results (SEER) Program, the patients with 5 or more years of survival after the diagnosis of small intestine cancer found to have 2 fold increase in risk of colon cancer. Similarly patients with colon cancer also found to have increased risk of small intestine cancer and these risks were amplified in patient whom primary cancer was diagnosed before 60 years of age [10]. Although there is an association between cancer of small and large bowel, surveillance EGD is not recommended at this time because of very low incidence.

According to Veen et al, it is extremely rare to have right sided colonic tumors causing delayed metastatic involvement of the duodenum after resection of the primary tumor [11]. Although very rare, cases of colon cancer with duodenal metastasis have been described in the literature along with the pathogenesis of metastasis to the duodenum [12]. Lymphatics from the right colon drain to the periduodenal lymph nodes leading to lymphatic spread from the colon to the duodenum. Also, the mesentery of the hepatic flexure of the colon lies in direct contact with the second portion of the duodenum, forming a pathway for metastatic spread by way of lymphatics [11-13]. Direct extension from the colon to the duodenum is also possible, but it’s less likely. Various primary neoplasms have their own distinct way of metastasis to duodenum, such as metastasis from lung, breast and melanoma spreads via blood and lymphatics while metastasis from colon, ovary and stomach spreads via peritoneal involvement [13].

Patients who underwent curative resection of colon should be followed up closely according to surveillance guidelines developed by various organizations. The National Comprehensive Cancer Network (NCCN) has developed guidelines for surveillance in patient with colorectal cancer which includes history and physical examination along with CEA measurement every 3 - 6 months for first 2 years and then every 6 months for total 5 years for stage II and III disease. The joint update of guidelines by the American Cancer Society and the US Multi-Society Task Force on Colorectal Cancer along with NCCN also recommends colonoscopy in 1 year after the curative resection of primary neoplasm for stages I through III disease [14, 15].

Because of vague presentation of duodenal carcinoma, there is delay of around 6 - 8 months between the first symptom occurrence and diagnosis [13]. Often the first presenting symptom is vague epigastric pain which starts or gets worse with eating. Other symptoms include weakness, fatigue, and weight loss. Once tumor gets enlarged, patient develops clinical manifestation of partial or complete obstruction such as nausea and vomiting. Occult gastrointestinal bleeding is also reported as presenting feature in the literature [1, 16, 17].

EGD and contrast studies are the first line for diagnosis of duodenal carcinoma in suspected cases as it allows determination of location, severity and length of involvement. EGD also allows taking biopsy of the lesion [16, 18]. As with any other cancer, histologic confirmation is required and patient also needs staging work up before starting treatment. Because of rarity of disease and very low incidence, there are no major studies comparing different treatments. If the patient is a candidate for surgery, aggressive surgery is the only curative option. Generally Adenocarcinoma involving first and second portion is treated with pancreatoduodenectomy while cancer involving third and fourth portion of duodenum requires duodenalsegmentectomy. Adjuvant radiotherapy and chemotherapy has also been used in selected cases [1, 13, 16]. Often patients with extensive metastatic disease receives only palliative and hospice care. The median age of survival for duodenal adenocarcinoma has been reported as 18 months with 5-year survival rate of 23% [13].

### Conclusion

Although rare, duodenal metastasis from colorectal cancer has been described in the literature. Recurrence of colon cancer is common, but metastasis to duodenum is very rare. Various primary neoplasms have their own distinct way of metastasis to duodenum. Lung cancer is the most common primary neoplasm for duodenal metastasis. Vague epigastric discomfort and pain is often first presentation. There is no specific presentation leading to diagnostic delay from the first symptom. Although association between cancer of small and large intestine has been documented, surveillance EGD is not recommended at this time in patient with colon cancer. Aggressive surgery is the only curative option.
